# Ultrasound guided femoral nerve block: An essential pain management modality in emergency settings for femur fractures

**DOI:** 10.4103/1658-354X.65127

**Published:** 2010

**Authors:** Dheeraj Kapoor, Sanjeev Palta

**Affiliations:** *Department of Anaesthesia, Government Medical College and Hospital, Chandigarh, India*

Sir,

Femur fractures are excruciating injuries that are frequently seen in patients requiring a trauma workup in the emergency room (ER) prior to definitive surgical management. These injuries are extremely painful and merit prompt attention to adequate analgesia and are considered by many as a key management aim.[1]

Femoral nerve block (FNB) can significantly decrease the acute pain of a diaphyseal or distal femoral fracture and fracture neck of femur. This analgesic modality can be administered safely in the hospital emergency department and in the prehospital settings prior to the operative intervention. Profound analgesia is obtained without the adverse effects associated with systemic intravenous analgesics (i.e., respiratory depression, hemodynamic effects, or obtundation of consciousness). This block is also very successfully used to facilitate positioning for placement of neuraxial block in the operating room (OR).[2]

We in our institute routinely administer ultrasound (Sonosite^®^ Titan^™^ with LHFL 38 high frequency 13-6 MHZ 38-mm linear array transducer) guided femoral nerve block prior to the operative intervention in femur fractures. We used out-of-plane needle insertion approach, by placing the insulated needle (5 cm, 22G) perpendicular to the transducer and ultrasound beam to inject local anesthetic (ropivacaine hydrochloride 0.75%) solution of mere 20 ml volume. We found that out-of-plane needle insertion approach is more convenient than the in-plane approach as the latter is more time consuming for alignment of needle with the ultrasound beam, which is often not possible in emergency situations. Moreover, if needed, catheter placement is easier with out-of-plane approach for prolonged analgesic requirements.

Conformation of the needle placement in peripheral nerve blocks is seen by many as a vital part of the procedure. In the emergency department, the use of a nerve stimulator for femoral nerve block would result in muscular contraction that would cause increased pain and would risk fracture displacement. Ultrasound guided FNB may be considered as a safe and an effective alternative to electrical nerve stimulation. Despite having a definitive advantage of clearly locating the nerves and negating the side effects caused by the nerve stimulator, the clarity of the image obtained can be obscured due to the presence of edema and subcutaneous air leading to block failure.[3]

Patients on emergency transport are prone to extreme stress reactions in painful conditions. Preclinically administered FNB can effectively decrease pain, anxiety and heart rate after femoral trauma.[4]

Ultrasound guided FNB can be used as an effective analgesic modality in patients with femur fractures thereby reducing post-traumatic stress disorder and the adverse effects of general anesthesia. This technique may decrease the onset time, improve the quality, reduce failure rate and permit a lower dose of local anesthetic.[5]

It is recommended that ultrasound guided FNB be considered as an important standard of care for femur fractures at emergency departments and in the OR prior to operative intervention. FNB can also be considered as an option for out-of-hospital analgesia for purposes of transportation and immobilization, provided it is administered by trained healthcare providers.[6]
